# Self-reported adult footwear and the risks of lower limb osteoarthritis: the GOAL case control study

**DOI:** 10.1186/1471-2474-15-308

**Published:** 2014-09-20

**Authors:** Daniel F McWilliams, Stella Muthuri, Kenneth R Muir, Rose A Maciewicz, Weiya Zhang, Michael Doherty

**Affiliations:** Division of Rheumatology, Orthopaedics and Dermatology, University of Nottingham, Nottingham, UK; AstraZeneca, London, UK; University of Warwick, Warwick, UK; Academic Rheumatology, Clinical Sciences Building, City Hospital, Nottingham, NG5 1PB UK

**Keywords:** Footwear, Osteoarthritis, Hip, Knee, Shoe

## Abstract

**Background:**

Biomechanical factors may play a role in osteoarthritis (OA) development and progression. Previous biomechanical studies have indicated that types of footwear may modulate forces across the knee joint, and high heeled womens’ shoes in particular are hypothesised to be detrimental to lower limb joint health. This analysis of data from a case control study investigated persistent users of different adult footwear for risks of knee and hip OA. Our underlying hypotheses were that high heeled, narrow heeled, and hard soled shoe types were putative risk factors for lower limb OA.

**Methods:**

Data on footwear were initially obtained from participants during the Genetics of Osteoarthritis and Lifestyle (GOAL) hospital-based, case control study using standardised interview-delivered questionnaires. An additional questionnaire was later sent to GOAL study participants to verify findings and to further investigate specific shoe use per decade of life. Persistent users of footwear types (high or narrow heel; sole thickness or hardness) were identified from early adulthood. Participants were grouped into single sex knee OA, hip OA or control groups. Adjusted odds ratios (aOR) and 95% confidence interval (CI) were calculated.

**Results:**

Univariate analysis of persistent users of women’s high heeled and narrow heeled shoes during early adulthood showed negative associations with knee OA and hip OA. After logistic regression, persistent narrow heel users were associated with less risk of OA (knee OA aOR 0.59, 95% CI 0.35 – 1.00 and hip aOR: 0.50, 95% CI 0.30 – 0.85), and other analyses were not statistically significant. Further analysis suggested that women with hip OA may have stopped wearing high and narrow heeled footwear to attenuate hip pain in early adulthood. Consistent associations between shoe soles and OA were not found.

**Conclusions:**

In general, persistent users of high and narrow heeled shoes during early adulthood had a negative association with knee or hip OA. This does not necessarily imply a causal relationship, as changing footwear during early adulthood to modulate index joint pain may provide a possible explanation. Despite the findings of previous biomechanical studies of high heels, we did not find a positive association between women’s shoes and lower limb osteoarthritis.

**Electronic supplementary material:**

The online version of this article (doi:10.1186/1471-2474-15-308) contains supplementary material, which is available to authorized users.

## Background

Osteoarthritis (OA) is a common chronic condition in the ageing population, and is responsible for pain and loss of function in a large number of sufferers[1]. Biomechanical factors play a role in OA progression[2, 3] and persistent exposure throughout life is thought to contribute to the development of OA[4].

Walking while wearing footwear acts to increase forces across the knee joint and hip joint compared to bare feet[5–7]. Therefore it is possible that different items of everyday footwear, which may differentially increase the forces across the joints, may confer different risks of lower limb OA in later life. Gait analysis studies indicate that ladies high heeled shoes increase the knee adduction moments across the knee joint[8, 9]. Neutrally-angled orthoses have also been associated with improved pain and function in patients with knee OA over 12 months in a clinical trial, although placebo response was not ruled out since they were the control group with some expectancy for improvement[10]. Similarly, improvements in pain and function scores were seen in both groups of a footwear clinical trial (high-end walking boots and “unstable” footwear)[11] and there is interest in developing shoes that reduce adduction moments across the knee[12]. A specially-designed hip replacement that was used to measure forces across the hip during gait in post-operative patients, found that very hard-soled shoes were responsible for the greatest force[5]. Changing footwear to soft, thick soled shoes, such as trainers, is often recommended for patients with OA[13], as it is a common sense intervention that is easy to implement. In patients with established OA, reduction of adduction moments across the joints appears to reduce the rate of progression[2, 14, 15]. Although high heeled shoes might be thought to increase the risk of lower limb OA[9], epidemiological studies of footwear and OA risk are rarely reported. One small case–control study did not find any elevated risk of knee OA in high heel wearing women[16].

In this study we have examined whether persistent users of ladies’ heels and different types of shoe sole in the GOAL study database were associated with risks of knee and hip OA. We hypothesised that high heels and narrow heels were risk factors for lower limb OA due to increased forces across the knee joint. We also hypothesised that hard soled shoes might result in greater impacts while walking.

## Methods

### GOAL study

Participants were men and women originally recruited in Nottingham, UK between 2002 and 2006 to participate in the Genetics of Osteoarthritis and Lifestyle (GOAL) case–control study[17–21]. This study was primarily designed to detect genetic associations with OA and interactions with lifestyle and environment. Approval for the GOAL study was obtained from the Nottingham Research Ethics Committee. All participants gave informed, written consent as per the Declaration of Helsinki. The research conformed to STROBE guidelines for reporting observational studies.

Cases of OA were recruited from hospital orthopaedic surgery lists and from a rheumatology OA clinic. All cases had been referred to the hospital with symptomatic, clinically severe knee or hip OA, and the majority had undergone total joint replacements within the previous 5 years. Subjects were excluded from the study if they had other major arthropathy (e.g., rheumatoid arthritis or ankylosing spondylitis), Paget’s disease of bone affecting the pelvis or femur, overt childhood hip disease (e.g., Legg-Calve’-Perthes, slipped femoral epiphysis, or severe acetabular dysplasia), total hip replacement due to trauma or avascular necrosis of the femoral head, or a terminal illness. Control subjects were recruited from lists of people who had been referred to hospital for intravenous urography (IVU) within the last 5 years. Individuals who had no radiographic evidence of hip OA on their screening IVU radiograph, no hip or knee symptoms or any other exclusions criteria were invited to participate. Cases and controls were further characterized by interview and examination. Height and weight were measured to calculate body mass index (BMI). Participants reported their body weight at age 20, for estimation of BMI using current height. Previous knee or hip injuries were self-reported. Occupational risk was scored for lifting, kneeling, squatting and heavy standing work on a scale of 0–5 as previously reported[19], and was dichotomised for use in multiple regression models (0 or 1–5).

### Self-reported footwear during adulthood

During the GOAL study our preliminary findings were from a standardised questionnaire delivered by a trained interviewer which included questions about footwear worn during work and while socialising or walking. Each occupation was recorded during the participant’s lifetime to date, and the usual shoe worn for that job was reported. The usual footwear worn while walking or socialising were also recorded for each decade of life. For the analyses, we selected age ranges that we expected to be mostly free from knee and hip OA, allowing footwear to modify risks of incident OA rather than OA progression. Predominant heel or sole was defined as the footwear worn during the participant’s main job and also while either socialising or walking. The age ranges for early adulthood exposure (21 – 30) and long-term adult use (21 – 50) years were each taken and predominant footwear use was estimated. The age periods were chosen as time frames that likely commenced before the aetiological time window for OA development.

In order to clarify and verify the initial findings, an additional questionnaire was sent to GOAL study participants in January 2008, which included questions asking for percentage use of shoe types during each decade of adulthood (Additional file1). 3022 questionnaires were sent out, 2172 replied and 1551 of those eligible answered the footwear questions[19]. Line drawings of different shoe types were provided as a guide for ladies’ heels (high, medium or low; and wide-based or narrow-based) and soles for both genders (thick or thin; and hard or soft). Participants provided a percentage estimate of how long they wore each type of footwear. Predominant footwear types were taken as >50% usage during ages 20 – 29 years (early adulthood) and 20–39 years (adulthood), as longer time periods gave small numbers of persistent heel users. As a secondary analysis, tertiles of percentage reported heel use (during each decade) were calculated to investigate any dose–response relationship with OA risk. Participants were also asked if they had changed shoe type due to pain, what decade they changed and where the pain was located.

### Statistics

Differences between continuous demographic variables were analysed using one-way ANOVA with post-hoc Bonferroni corrections. Pearson’s X^2^ was used to examine dichotomous demographic data between groups. Odds ratios (OR), adjusted OR (aOR) and 95% confidence intervals (CI) were calculated to estimate risk of OA. For the purposes of adjustment, age was split into tertiles and BMI at age 20 was classified as <25 or ≥25. Occupational risk during longest-held job within the exposure time window and any previous index joint injury were also adjusted for (Yes/No for both). Analyses were undertaken separately for men and women due to their different uses of footwear. The reference groups for analyses were flat/low/medium heels, soft soles and wide heels. Thin soles were selected for a reference group because they were the most abundant, when compared to thick soles. Statistical significance was taken as p < 0.05 and analysis was performed using SPSS v14 (SPSS Inc, Chicago, USA).

## Results

Demographics for the GOAL study, which provided footwear data for the initial analyses, and demographics for the subgroup that responded to the additional footwear questionnaire are shown in Table 1. Both groups were similar for major characteristics.

**Table 1:** **Study demographics**

	GOAL interview footwear questions			Responders: Additional footwear questionnaire			Non-responders: Additional footwear questionnaire		
	Control	Knee OA	Hip OA	Control	Knee OA	Hip OA	Control	Knee OA	Hip OA
N=	1122	1007	1042	597	471	483	525	536	559
%Female	49%	48%	50%	47%	48%	49%	47%	49%	53%
Age	63 (9)	**68** (7)**	**68** (7)**	64 (8)	**67** (7)**	**67** (7)**	65 (9)	**69** (7)**	**68** (7)**
BMI	27 (5)	**31** (5)**	**29** (5)**	28 (5)	**31** (6)**	**29** (5)**	27 (5)	**31** (5)**	**29** (5)**
BMI in 20’s	22.5 (5.5)	**24** (4)**	**23** (4)**	23 (6)	**24** (3)**	**23** (4)**	22 (3)	**24** (4)**	**24** (4)**
Knee injury	17%	32%**	17%	18%	34%**	19%	16%	30%**	14%
Hip injury	2%	3%	7%**	2%	3%	10%**	2%	3%	5%*

The demographics of the responders to the footwear questions from the additional GOAL questionnaire are shown. They were similar to the demographics of the main GOAL study (published elsewhere[19]). Mean (s.d.) or percentage prevalence of characteristics within study groups. **-p < 0.01, *-p < 0.05 vs controls. Statistical significance is highlighted in **bold**.

Our preliminary findings on footwear were derived from the data collected by trained interviewers, where interviews were performed for all participants, and predominant heel type was estimated for the main work shoe plus that worn while either socialising or walking. A negative relationship between OA and high/narrow heeled shoes was observed for the age ranges 21 – 30 and 21 – 50 years (Table 2). After adjustments for well established OA risk factors, a significant association remained with narrow heels and lower limb OA (Table 2).

**Table 2:** **Work and social/walking heels from the GOAL interview**

GOAL interview: Female heel types		Groups			Risk of knee OA		Risk of hip OA	
		Control	Knee OA	Hip OA	Univariate	Adjusted	Univariate	Adjusted
21-30 yrs	Low or flat (ref)	374	379	401	0.52 (0.35 - 0.78)**	0.76 (0.46 - 1.27)	0.55 (0.37 - 0.81)**	0.82 (0.44 - 1.17)
	High	78	41	46				
	Wide (ref)	393	394	408	0.78 (0.56 - 1.08)	0.99 (0.65 - 1.51)	0.69 (0.48 - 0.93)*	0.82 (0.54 - 1.24)
	Narrow	101	79	70				
21-50 yrs	Low or flat (ref)	441	447	455	0.55 (0.33 - 0.90)*	0.53 (0.27 - 1.01)	0.43 (0.26 - 0.74)**	0.64 (0.34 - 1.18)
	High	47	26	21				
	Wide (ref)	410	429	432	0.69 (0.45 - 1.04)	0.59 (0.35 - 1.00)	0.54 (0.35 - 0.84)**	0.50 (0.30 - 0.85)*
	Narrow	60	43	34				

Data taken from the GOAL study interview. Predominant footwear (during work and also socialising or walking) and risks of OA (adjustments performed for age, BMI in 20’s, occupational risk and previous injury to index joint. **-p < 0.01, *-p < 0.05. Reference groups (ref) indicated within table. Statistical significance is highlighted in **bold**.

The data from the additional questionnaire, sent out at a later date, were analysed for women’s heel users to determine whether a similar pattern of negative associations were seen. Univariate analyses confirmed that high heel users had a lower risk of knee or hip OA than the pooled group of low/medium heel users (the risks of OA in low and medium heel users were similar). Adjustments for confounders (other OA risk factors) yielded a borderline, but significant, association for hip OA risk and high heels (Table 3). Narrow heel bases also had the negative association with knee OA confirmed after univariate analysis (Table 3), but not after logistic regression. The sample size of responders from the additional questionnaire was lower than the interviewer-led data (Tables 1,2 and3).

**Table 3:** **Women’s heels and risks of OA from the additional questionnaire**

Additional questionnaire: Female heel types		Groups			Knee OA		Hip OA	
		Control	Knee OA	Hip OA	Univariate	Adjusted	Univariate	Adjusted
20-29 yrs	Low or medium (ref)	96	84	101	0.56 (0.32 - 0.98)*	0.74 (0.40 - 1.37)	0.50 (0.29 - 0.87)*	0.55 (0.31 - 0.99)*
	High	51	25	27				
	Wide (ref)	57	55	72	0.54 (0.31 - 0.96)*	1.02 (0.57 - 1.82)	0.47 (0.27 - 0.80)**	0.68 (0.40 - 1.16)
	Narrow	61	32	36				
20-39 yrs	Low or medium (ref)	90	82	96	0.64 (0.31 - 1.32)	0.77 (0.35 - 1.71)	0.63 (0.31 - 1.25)	0.71 (0.33 - 1.55)
	High	24	14	16				
	Wide (ref)	93	57	76	0.62 (0.37 - 1.03)	0.86 (0.45 - 1.63)	0.50 (0.31 - 0.81)**	0.62 (0.34 - 1.13)
	Narrow	82	46	49				

Persistent users of women’s heels in early adulthood (20–39 years) and adulthood (20 – 39 years) are presented. Numbers of users from ages 20–49 were too low for meaningful analysis. OR (95% CI) and aOR (adjusted for age, BMI in 20’s, occupational risk and previous injury to index joint) are shown. Persistent users were defined as >50% user in every decade, and variable users were not assessed. **-p < 0.01, *-p < 0.05. Reference groups (ref) indicated within table. Statistical significance is highlighted in **bold**.

Total percentage use of high and narrow heels in the age range 20–29 was calculated from responses to the additional questionnaire. The percentage use of high or narrow heels was also negatively associated with OA risk after univariate analysis. Tertiles of high heel use were negatively associated with hip OA after univariate analysis by logistic regression (OR 0.76, 95% CI 0.61 – 0.94; P-trend = 0.011) but were negative and non-significant after adjustments for age and BMI (aOR 0.82, 95% CI 0.65 – 1.03; P-trend = 0.088). Narrow heel use from ages 20–29 showed a similar pattern with hip OA (univariate analysis: OR 0.70, 95% CI 0.55 – 0.88: P-trend = 0.002; and adjusted analysis: aOR 0.83, 95% CI 0.63 – 1.04; P-trend = 0.098). It was noted that persistent high heel and narrow heel users were less likely to have experienced occupational risk factors for OA than the flatter heel group (10% vs 33%; p < 0.001; and 13% vs 31%; p < 0.001 respectively).

Adjusted analysis showed a borderline association between persistent thick soled shoe users and knee OA in females (Table 4A) but not males (Table 4B). After adjustments for confounders, our analyses did not reveal any significant associations between shoe soles and OA in women or men (Table 4A/B).

**Table 4:** **Sole characteristics and risk of OA from the additional questionnaire**

A								
Female	Sole	Groups			Knee OA		Hip OA	
		Control	Knee OA	Hip OA	Univariate	Adjusted	Univariate	Adjusted
20-29 yrs	Thin (ref)	142	99	106	1.62 (0.94 - 2.78)	1.29 (0.70 - 2.40)	1.17 (0.66 - 2.07)	1.12 (0.59 - 2.11)
	Thick	31	35	27				
	Soft (ref)	30	37	23	0.62 (0.35 - 1.09)	0.55 (0.30 - 1.02)	1.05 (0.56 - 1.94)	1.12 (0.57 - 2.20)
	Hard	101	77	81				
20-39 yrs	Thin (ref)	121	79	92	2.23 (1.21 - 4.11)*	1.98 (1.00 - 3.91)	1.14 (0.58 - 2.22)	1.01 (0.48 - 2.12)
	Thick	22	32	19				
	Soft (ref)	26	30	21	0.76 (0.41 - 1.43)	0.66 (0.33 - 1.30)	1.15 (0.59 - 2.24)	1.09 (0.52 - 2.28)
	Hard	67	59	62				
**B**								
**Male**	**Sole**	**Groups**			**Knee OA**		**Hip OA**	
		**Control**	**Knee OA**	**Hip OA**	**Univariate**	**Adjusted**	**Univariate**	**Adjusted**
20-29 yrs	Thin (ref)	62	36	55	1.34 (0.84 - 2.15)	1.13 (0.67 - 1.92)	0.74 (0.48 - 1.15)	0.69 (0.44 - 1.10)
	Thick	163	127	107				
	Soft (ref)	39	18	24	1.51 (0.83 - 2.75)	1.82 (0.94 - 3.52)	1.16 (0.67 - 2.02)	1.06 (0.60 - 1.89)
	Hard	194	135	139				
20-39 yrs	Thin (ref)	52	32	51	1.29 (0.78 - 2.14)	1.11 (0.63 - 1.95)	0.69 (0.43 - 1.09)	0.65 (0.40 - 1.06)
	Thick	147	117	99				
	Soft (ref)	33	17	20	1.39 (0.74 - 2.61)	1.65 (0.82 - 3.32)	1.21 (0.66 - 2.21)	1.03 (0.55 - 1.93)
	Hard	169	121	124				

Persistent female and male users of certain types of shoe sole in their 20’s and through early adulthood are presented for female (A) and male (B) participants in the GOAL study. OR (95% CI) and aOR (adjusted for age, BMI in 20’s, occupational risk and previous injury to index joint) are shown. Persistent users were defined as >50% user in every decade, and variable users were not assessed. *-p < 0.05. Reference (ref) groups indicated within table.Statistical significance is highlighted in **bold**.

Additionally, we examined the risks of OA in those that reported changing footwear due to pain in early adulthood. This may have been an important modulator of the exposure and the risks. Those who reported changing footwear due to knee pain were more likely to be within the knee OA study group than the others (X^2^ = 208, p <0.001). The same was also observed with changing footwear due to hip pain and being part of the hip OA study group (X^2^ = 225, p < 0.001). Those who changed footwear due to foot pain were distributed similarly across the groups (X^2^ = 3.2, p = 0.201). Conversely, within the hip OA group, the high or narrow heel users (in their 20’s) were more likely to have changed footwear due to hip pain (Table 5) than flatter/wider heel users. Within the knee OA group, high or narrow heels in women were not associated significantly with changing footwear due to knee pain (Table 5).

**Table 5:** **Associations between changing footwear due to joint pain and OA**

		Footwear in 20s	OR (95% CI)	
			Female	Male
Knee OA cases	Changed footwear due to knee pain (20-39y)	High heels vs Low/medium (ref)	1.78 (0.67 - 4.69)	N/D
		Narrow heels vs Wide (ref)	1.49 (0.68 - 3.27)	N/D
		Thick soles vs Thin (ref)	1.73 (0.78 - 3.81)	0.77 (0.28 - 2.13)
		Hard soles vs Soft (ref)	0.87 (0.39 - 1.91)	1.23 (0.26 - 5.81)
Hip OA cases	Changed footwear due to hip pain (20-39y)	High heels vs Low/medium (ref)	3.60 (1.45 - 8.94)	N/D
		Narrow heels vs Wide (ref)	2.64 (1.27 - 5.50)	N/D
		Thick soles vs Thin (ref)	0.36 (0.14 - 0.91)	3.01 (0.64 - 14.10)
		Hard soles vs Soft (ref)	1.61 (0.62 - 4.23)	0.67 (0.18 - 2.58)

Univariate *post hoc* subgroup analysis of changing footwear due to pain. Data taken from the additional questionnaire. Data from hip OA cases that changed footwear due to hip pain, and knee OA cases that changed footwear due to knee pain are shown. Participants that reported changing footwear due to knee or hip pain between the ages of 20 and 39 are shown with the footwear they reported using persistently during their 20’s. Persistent users were defined as >50% user per decade, and variable users were not assessed. Unadjusted OR and 95% CI are shown for the association between the site of pain and the type of footwear used. N/D – analysis not done. Reference groups (ref) indicated within table.

## Discussion

The data from two separate footwear questionnaires delivered to the same study group showed negative associations between high heeled and narrow heeled women’s shoes and lower limb OA, although some of the associations were explained by confounders. The two datasets appeared consistent with regards to the major univariate findings of negative associations between persistent users of high/narrow womens’ heels and lower limb OA.

As high heeled shoes increase forces across the knee during gait[8, 9], they are actually candidates to be positive risk factors for OA. Narrow heels in women’s shoes could confer more instability than wide heels, and therefore alter biomechanical forces across the joints. However, our study and also one by Dawson *et al.*[16] reported no positive association between female high heel users and the development of OA. One possible interpretation of the negative association is that those who chose to be persistent users of high heels were from lower risk groups, such as those with lower BMI or less occupational risks. Women with strenuous jobs would be unlikely to wear high or narrow heeled shoes while working. Persistent high/narrow heel users may also have been from a lower risk group of women without hip pain during early adulthood. A subset of women from our *post hoc* analysis within the hip OA group reported wearing high heeled shoes but abandoning them due to hip pain. The negative association between OA and high heels might be explained by regular users over long time periods being those who were less likely to experience index joint pains. In other words, they could be a self-selecting low-risk subgroup. Indeed, changing footwear was reported as a response to index joint pain. Some studies have reported improved joint pain after changing footwear[11, 22]. As many women gave up wearing high heels as their predominant shoes, our statistical analysis over longer time periods was less powerful. There are likely to be a large number of reasons to stop wearing high heeled shoes, and pain is just one possibility.

Thick-soled shoes were hypothesised to be protective against OA, as they would absorb impact and lessen the load transferred through the lower limbs. Instead, there were no significant associations with OA. No consistent relationship with increasing time of use was seen between hard and soft soles and OA.

Most of the cases in this study had clinically-severe, and often end-stage, OA and most had joints replaced when entering the GOAL study. This study relied upon the recall of participants, and this is an important source of bias. Comparing patterns of persistent footwear use in severe OA cases with asymptomatic controls without detectable radiographic OA will have maximised the measured risks. The associations between milder cases of OA and footwear may differ from those in our study. Our findings about persistent users would also suggest that no significant association with lower limb OA would be detected in a study of heterogeneous (non-persistent) users of different types of adult footwear, certainly after adjustments for important confounders. The selection methodology for persistent users differed between the 2 questionnaires. For the GOAL interview, we used work, walking and social shoes from early adulthood. However, for the additional questionnaire we asked about percentage use per decade of life. The analyses of the additional questionnaire may have included a group of shoe users with lower exposure (more variety of shoe types) than those that wore high heels predominantly while working/walking/socialising (more people were classified as high heel users in the additional questionnaire group). The demographics of the responder and non-responder groups to the additional questionnaire were similar, implying that this was not a major source of bias.

## Conclusions

In conclusion, the role of common footwear types in the development of knee and hip OA appears to be limited, with no positive associations reported in women or men. The negative associations that we report are borderline statistically significant, and might be explained by unknown confounders or by a self-selecting group of low-risk people. However, prospective long-term studies will be more useful in determining these relationships.

## Electronic supplementary material

Additional file 1:Additional questionnaire sent to GOAL study participants.(PDF 1 MB)

## Abbreviations

IVU: Intravenous urography
OA: Osteoarthritis
OR: Odds ratio
aOR: Adjusted odds ratio
CI: Confidence interval
IVU: Intravenous urography
BMI: Body mass index
GOAL: Genetics Osteoarthritis and Lifestyle study.
