# Supplementary material for: Self-reported adult footwear and the risks of lower limb osteoarthritis: the GOAL case control study
Source: BMC Musculoskelet Disord. 2014 Sep 20;15:308. doi: 10.1186/1471-2474-15-308 (PMC4190490; doi:10.1186/1471-2474-15-308)
Supplement: Supplementary file 1 — Additional file 1:Additional questionnaire sent to GOAL study participants.(PDF 1 MB) [file 12891_2014_2254_MOESM1_ESM.pdf]

## SECTION 9
## Footwear

You may remember that when you participated in the GOAL Study, we asked you about the types of shoes that you wore during leisure and work activities. We now wish to investigate this further, since it appears that certain features of a shoe may relate to the development of pain and osteoarthritis of the foot, knee and hip.

This section is split into three parts to ask you about different elements of the shoes you have worn during different decades of your life. The three separate parts of the shoe we are interested in are: **sole**, **heel** and **forefoot.** When looking at the pictures and descriptions below, **please only consider the element we are asking you to focus on – that is, the type of sole, heel or forefoot that you wore.**

For each section, we would like you to think about an average week in each decade and estimate what **proportion of the time that you were wearing shoes (e.g. shoes worn for work, going out socially, for recreational activities etc) did you wear shoes with each of the listed characteristics.** Within each section, you may choose more than one type of shoe for each decade. The following table is given as an example of a patient who is 45 years old:

**EXAMPLE**

| | EACH DECADE | | | | |
|---|---|---|---|---|---|
| **SOLE TYPE** | **20s** | **30s** | **40s** | **50s** | **60s** |
| Thick hard sole | 50% | 50% | 40% | | |
| Thick soft sole | 30% | 40% | 40% | | |

As you will see, the example above shows that this person in their 20s wore a 'thick hard sole' for 50% of the time that they wore shoes and a 'thick soft sole' for 30% of the time that they wore shoes.

**MEN AND WOMEN**

**For EACH DECADE please estimate what proportion of time that you were wearing shoes did you wear shoes with each of the following characteristics:**

| | EACH DECADE | | | | |
|---|---|---|---|---|---|
| **SOLE TYPE** | **20s** | **30s** | **40s** | **50s** | **60s** |
| Thick hard sole | | | | | |
| Thick soft sole | | | | | |
| Thin hard sole | | | | | |
| Thin soft sole | | | | | |

**MEN –** Please answer the following set of questions below relating to heel type and forefoot type. You may choose more than one type of shoe heel or forefoot for each decade.

(**WOMEN –** Please move to *Page 14* to answer questions relating to heel type and forefoot type).

## MEN ONLY

**For EACH DECADE please estimate what proportion of time that you were wearing shoes did you wear shoes with each of the following characteristics:**

Please complete each line as shown in the example on page 12.

| HEEL TYPE | 20s | 30s | 40s | 50s | 60s |
|---|---|---|---|---|---|
| 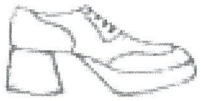 High heel, broad base | | | | | |
| 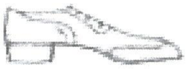 Medium heel, broad base | | | | | |
| 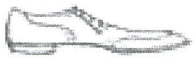 Low heel, broad base | | | | | |

| FOREFOOT TYPE | 20s | 30s | 40s | 50s | 60s |
|---|---|---|---|---|---|
| 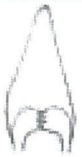 Narrow forefoot | | | | | |
| 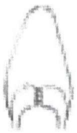 Medium narrow forefoot | | | | | |
| 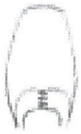 Broad forefoot | | | | | |
| 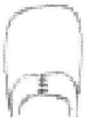 Very broad forefoot | | | | | |

*Please move to page 15*

## WOMEN ONLY

**For EACH DECADE please estimate what proportion of time that you were wearing shoes did you wear shoes with each of the following characteristics:**
(You may choose more than one type of shoe heel or forefoot for each decade)

Please complete each line as shown in the example on page 12.

| HEEL TYPE | 20s | 30s | 40s | 50s | 60s |
|---|---|---|---|---|---|
| 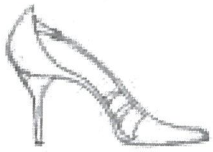 High heel, narrow base | | | | | |
| 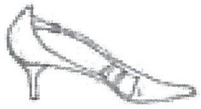 Medium heel, narrow base | | | | | |
| 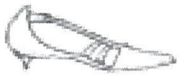 Low heel, narrow base | | | | | |
| 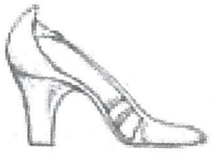 High heel, broad base | | | | | |
| 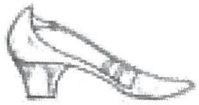 Medium heel, broad base | | | | | |
| 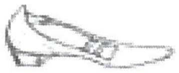 Low heel, broad base | | | | | |

## WOMEN ONLY (…….continued)

| FOREFOOT TYPE | 20s | 30s | 40s | 50s | 60s |
|---|---|---|---|---|---|
| 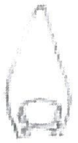 Narrow forefoot | | | | | |
| 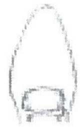 Medium narrow forefoot | | | | | |
| 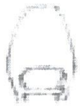 Broad forefoot | | | | | |
| 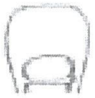 Very broad forefoot | | | | | |

## MEN AND WOMEN

We are interested to know whether you may have changed the types of sole, heel or forefoot you wore because of pain in your foot, knee, hip or somewhere else in your body. If you did change any of the elements of your shoe because of pain, please specify in which decade you changed them because of pain, by placing a tick (✓) in the appropriate box. *If you did change type of the sole, heel or forefoot, but for other reasons than pain, please do not place a tick.*

| | Each decade | | | | |
|---|---|---|---|---|---|
| | **20s** | **30s** | **40s** | **50s** | **60s** |
| **Sole** | | | | | |
| **Heel** | | | | | |
| **Forefoot** | | | | | |

If you did change your footwear because of pain, please state where you experienced this pain (e.g. knee, hip, back, forefoot, ankle)?

……………………………………………………………………………………………………………

……………………………………………………………………………………………………………
